# The *in vitro* antisickling and antioxidant effects of aqueous extracts *Zanthoxyllum heitzii* on sickle cell disorder

**DOI:** 10.1186/1472-6882-13-162

**Published:** 2013-07-06

**Authors:** Nanfack Pauline, Biapa Nya Prosper Cabral, Pieme Constant Anatole, Ama Moor Vicky Jocelyne, Moukette Bruno, Ngogang Yonkeu Jeanne

**Affiliations:** 1 Department of Biochemistry and Physiological Sciences, Faculty of Medicine and Biomedical Sciences, University of Yaoundé I, PO Box 1364, Yaounde, Cameroon; 2 Department of Biochemistry, Faculty of Sciences, University of Dschang, Cameroon, PO Box 67, Dschang, Cameroon; 3 University Center teaching hospital, Yaoundé, Cameroon

**Keywords:** Sickling cells, Reversibility, Antioxidant, Hemolysis, *Zanthoxyllum heitzii*

## Abstract

**Background:**

Several plant extracts from Rutaceae family are currently used to the management of sickle cell disorder (SCD) in the African. Few reports have shown that extracts from *Zanthoxyllum* or *Fagara* genus demonstrated anti-sickling property. This study investigates the *in vitro* antisickling and antioxidant properties of extracts from *Zanthoxyllum heitzii*.

**Methods:**

The sickling of red blood cells (RBCs) was induced using sodium metabisulfite (2%) followed by treatment with extracts at different concentrations. The osmotic fragility tests permits to explore the effect of *Z. heitzii* extracts on haemoglobin S solubility and sickle cells membrane stability. For each extract, qualitative phytochemical tests were used to identify the presence of alkaloids, tannins, saponins, flavonoids, glycosides and phenols, while some quantitative methods such as Folin, Ferric Reducing Antioxidant Power (FRAP) and diphenyl 1, 2 picryl hydrazyl (DPPH) were used to determine the antioxidant potential of these extracts.

**Results:**

Sodium metabisulphite increased the sickling of RBCs from 29.62 to 55.46% during 2 h. Treatment of sickling cells with extracts at different concentrations showed that a decrease of the percentage of sickling cells was found in both induced and non induced sickling cells. The fruits extract of *Z. heitzii* demonstrated the best anti-sickling property. The same extract at 250 μg/mL showed the best membrane cell stability compared to others. All the extracts revealed an antioxidant and anti-radical activities although lesser compared to the standard.

**Conclusion:**

The fruit extract of *Z. Heitzii* demonstrated the most significant antisickling effect with a potential for use in the clinical management of SCD.

## Background

Sickle-cell disorder (SCD), or sickle-cell anaemia is an autosomal recessive genetic blood disorder with overdominance, characterized by red blood cells that assume an abnormal, rigid, sickle shape and known to be one of the diseases afflicting the population living mostly in Africa, South America and Asia. It also occurs in other ethnic groups, including people who are of Mediterranean and Middle Eastern descent. In Cameroon, the carrier (AS/AC) rate is estimated between 20-25% while the reported prevalence of SCD is between 2-3% [1], and the disease is tending to become a public health problem. As a genetic hereditary disease, no specific drugs are yet available; however several treatments have been investigated: (1) medullar transplantation is not only expensive but also faces incompatibility problems; (2) various proposed drugs (hydroxyurea, piracetam, calcium antagonists) tested against this disease for inhibition of hemoglobin S polymerization inside erythrocytes in order to increase the foetal hemoglobin rate (HbF) or decrease the sickling are toxic especially for a long time of use [2,3]. Considering all genetic disorders, the debilitating effect, the cost of managing the SCD and the greater quantities of O^2–^, H_2_O_2_, •OH produced from sickle RBCs than do normal RBCs [4], research has been on-going to determine the efficacy of the use of natural products such as medicinal plants, nutritional complement against sickle cell disorder.

Some researchers have argued in favor of nutrition in the management of sickle cell disorder [5]. The use of traditional medicine can’t fade out in the treatment and management of an array of diseases in the African continent. Several studies demonstrated the anti-sickling property of various extracts of Rutaceae family among them *Zanthoxyllum macrophyla* and *Fagara zanthoxyloides*[6]. The roots of *F. zanthoxyloides* has been used in folk medicine during sickle cell crisis and also there is recent scientific reports its antisickling activity [7]. Various molecules with anti-sickling property such as divanilloylquinic acids, vanillic acid, p-hydroxy benzoic acid, p-fluoro benzoic acid, 2-dihyroxymethyl benzoic acid have been isolated from these plants [8,9].

*Zanthoxylum heitzii* (Aubrev. & Pellegr) or *Fagara heitzii* is a plant from Rutaceae family. The representatives of this family are distributed in 150 genera and 1500 species commonly found in tropical and warm temperate regions of the world [10,11]. It is a family of great importance due to its uses in perfumery, food industry and management of diseases by the Traditional Health Practitioners. *Zanthoxyllum heitzii* is one of the cultivar of *Fagara xanthozyloïdes* found in the West region of Cameroon. Its fruits is used as a spice for the preparation of “nkui” and “Nah poh”, two dishes of Cameoon [12]. It is also used as medicinal plant in central Africa for the treatment of many diseases such as cancer, syphilis, malaria, cardiac palpitations, uro-genital affections [13,14]. Several molecules were isolated from the stem bark of *Z. heitzii* among them, two amides: heitziamide A and heitziamide B and two phenylethanoids: heitziethanoid A and heitziethanoid B and s trans-fagaramide, arnottianamide, iso-c fagarine, iso-skimmianine, arctigenin methyl ether, savinin, (+)-eudesmin, (+)-sesamin, lupeol, lupeone, b-sitosterol, stigmasterol and stigmasterol-3-O-β-D-glucopyranoside [15]. The bark of *Z. heitzii* is used as insecticides and revealed some activities against cadiac affections [16]. Fagaricine, an aqueous extract formulation from root of *Z. heitzii* was demonstrated as an immune-restorative phytomedicine to treat immunodeficiency [17].

However, no studies have yet been carried out to investigate the antisickling and antioxidant properties of the various parts of this plant. The present study was performed with the aim to investigate the antioxidant property of *Z. heitzii* extracts, their effects on the sickling cells and on erythrocyte membrane stabilizing activities.

## Methods

### Plant materials and collection

Leaves, roots, fruits and stems barks of *Zanthoxylum heitzii* (Rutaceae) collected on the 30 June 2010 at Batchingou in the west of Cameroon and identified under the reference number 1441/H.N.C at the National Herbarium of Cameroon where the voucher specimen was deposited.

### Extraction of the plant material

Air-dried leaves, fruits, roots and stems of *Z. heitzii* were ground and an aliquot (150 g) was extracted by maceration (72 h) in 1.5 L of water as solvent. The same procedure was repeated with residues and the mixture was filtered and concentrated to dryness. The extract was stored at 4°C in freeze - dried form and used for the study.

### Collection of blood samples

The blood samples used in the evaluation of the anti-sickling activity of the plant extracts in this study were taken from patients between 19 and 31 years old known to have sickle cell disease, attending the Central hospital of Yaoundé . All these patients were confirmed regarding their SS status using the electrophoresis test. The blood samples were collected in the sodium EDTA tubes and stored for the experiment. A written informed consent was read and signed by all the patients participating in the study. All the research procedures have received the approval of Cameroon National Ethics Committee reference number 181/CNE/SE/2011 signed by Pr Lazare Kaptue, the president of the committee.

### Erythrocyte membrane stability activity

The osmotic fragility of erythrocytes measures the membrane stabilizing effect of the extracts in osmotic stress/hypotonic lysis incubation. To 10 mL reaction vessel containing 4 mL of different concentrations (0.00 - 0.85%) of buffered saline with pH of 7.4, 1 ml of each extract (250 μg/mL) and 0.05 ml SS-RBC blood were added. The mixture was incubated at room temperature (25°C) for 24 h and then centrifuged at 3000 rpm for 15 min. The optical density of the supernatant was read at 540 nm against blank made of 0.85% buffered saline concentration [18]. The mean corpuscular fragility (determined from the concentration of saline causing 50% haemolysis of the RBC) was obtained from a plot of lysis (%) *versus* NaCl concentration.

### Antisickling activity

#### In vitro induction of sickling

Five milliliters blood samples obtained from patients were centrifuged at 5,000 rpm for 10 min in saline thrice to obtain the RBC which were then resuspended in normal saline and used for the analysis according to the method described [19]. 100 μL of SS blood cell suspensions were mixed with 100 μL the presence of 2% sodium metabisulphite solution and incubated at 37°C. The time course of the sickling of SS erythrocytes was analyzed microscopically. The number of cell was counted every one hour and the percentage of sickling cells was calculated using the formula: (%) Sickling = Number of sickling cells × 100/total cells.

#### In vitro anti-sickling activity of the extracts

A serial of different concentrations of aqueous extracts *Z. heitzii* were prepared in the saline solution. For the assay 100 μL of SS-RBC pre-incubated with 2% Na_2_S_2_O_5_ was added to 100 μl of solution of different extracts for final concentration of 250, 500 and 1000 μg/mL. Each mixture was incubated at 37°C for 2 h (time necessary to obtain maximun sickling). After incubation, 10 μL of the mixture was diluted 100 times. A drop of each sample was examined under the oil immersion light microscope and both sickled cells and total cells were counted from five different fields of view across the slide. For the negative control, the solution containing the extract was replaced by the saline solution. The percentage of sickling was calculated using the formula: % of sickling = number of sickling cells × 100/total cells.

For the reversibility assay, freshly collected HbSS blood was diluted in 1:1 ratio with 0.9% normal saline (negative control) or test solution containing different extracts for final concentration of 250 μg/mL. The experiment was followed as mentioned above and the percentage of sickling cells was calculated.

### Antioxidant and phytochemical screening

The antiradical activity of the plant extract was examined based on the scavenging effect of the stable DPPH and hydroxyl free radical activity as well as the determination of power reducing and antioxidant capacity antivities.

#### Scavenging Activity of DPPH Radical

The DPPH free radical scavenging assay was carried out for the evaluation of the antioxidant activity [20]. Briefly, in 3 mL of each diluted extract, 1 ml of methanol solution of DPPH 0.1 mM was added. The mixture was kept in the dark at room temperature for 30 min and the absorbance was measured at 517 nm against a blank. The following equation was used to determine the percentage of the radical scavenging activity of each extract.

Percentage of radical scavenging activity = [(OD control - OD sample)/OD control] × 100 The IC_50_ value (μg/mL) is the effective concentration at which DPPH radicals were scavenged. by 50% and the value was obtained by interpolation from linear regression analysis.

#### Hydroxyl radical scavenging activity

The scavenging activity of the extract on hydroxyl radical was measured according to a previously described method [21]. In 1.5 mL of each diluted extract, 60 μL of FeCl_3_ (1 mM), 90 μL of 1,10- Phenanthroline (1 mM), 2.4 ml of 0.2 M phosphate buffer, pH 7.8 and 150 μL of H_2_O_2_ (0.17 M) were added respectively. The mixture was then homogenized and incubated at room temperature for 5 min. The absorbance was read at 560 nm against the blank. The percentage of the radical scavenging activity of each extract was calculated from the equation below:

Percentage of radical scavenging activity = [(OD control - OD sample)/OD control] × 100. The extract concentration providing 50% inhibition (IC_50_) was calculated and obtained by interpolation from linear regression analysis.

#### Reducing power assay

One milliliter of different concentration of extract (20-80-120-240 μg/mL) diluted in distilled water was mixed with 2.5 mL of phosphate buffer (0.2 M, pH 6.6) and 2.5 mL of potassium ferrocyanide (1%). The mixture was incubated at 50°C for 20 min. Aliquot (2.5 mL) of trichloroacetic acid (10%) was added to the mixture and centrifuged at 3000 rpm for 10 min. The upper layer of the solution (2.5 mL) was mixed with 2.5 mL of distilled water and 0.5 mL of FeCl_3_ (0.1%). The increased absorbance was measured at 700 nm against the blank indicates the increasing of the reducing power [22].

#### Total antioxidant activity by ferric reducing antioxidant power assay (FRAP)

The FRAP method was used to determine the total antioxidant activity which measures the reduction of ferric ion to the ferrous form in the presence of antioxidant compounds [23]. The fresh FRAP reagent consist of 500 mL of acetate buffer (300 mM pH 3, 6), 50 mL of 2, 4, 6- Tri (2- pyridyl)-s-triazin (TPTZ) (10 mM), and 50 mL of FeCl_3_ · 6H_2_O (50 mM). For the assay, 75 μL of each extract were mixed with 2 ml of FRAP reagent and the optical density was read after 2 min at 593 nm against the blank.

The standard procedure was used for screening *Z. heitzii* extract for the presence of alkaloids, tannins, saponins, flavonoids, glycosides and phenols [23].

### Statistical analysis

Each test was performed in triplicate and the results were expressed as mean ± standard deviation. The Kruskal-Wallis non parametric test followed by a post hoc Dunnet T_3_ (p < 0.05) was used to analyze the antioxidant capacity, total phenols content as well as the radical scavenging activity of each extract. The correlation between antioxidant capacity and total phenols content was established using the Pearson product moment correlation. The IC _50_ was determined with the multiple regression analysis. The software SPSS version 10.1 for Windows was used for statistical analysis.

## Results

**Figure 1:**
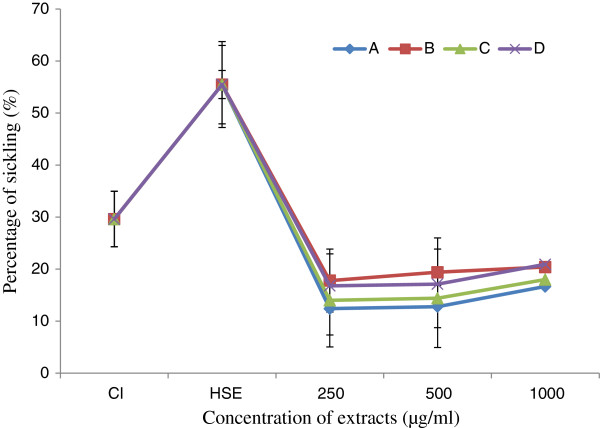
**Effect of sodium metabisulfite (2%) and extracts on sickling cells.** Cl: Initial sickling percentage, HSE: Sickling percentage after induction of with metabisulfite; **(A)**: Fruits; **(B)**: Leaves; **(C)**: Root/bark; **(D)**: Root wood.

The yields of extraction are between 3.20 and 5.78% depending on the part of the plant used (Table 1). The extract of leaves from *Z. heitzii* has a higher extraction yield compared to other. The result of induction of sickling with sodium metabisulphite (2%) shows an increase in sickling from 29.62 to 55.46% (Figure 1). Sodium metabisulphite (2%) induced sickling of erythrocytes with an average induction rate of 25.84% after 2 h of incubation.

**Table 1:** **Antioxidant and antiradical properties of the plant extract of *Z. heitzii***

***Part of plant studied***	***Extraction yield (%)***	***In vitro antiradical and antioxidant of the extract***			
		**IC**_**50 **_**(μg/mL)**		**Antioxidant Properties (at 500 μg/mL of extract)**	
		**DPPH**^**•**^	^**•**^**OH**	**Reducing power (Optical density at 700 nm)**	**Antioxidant potential (mgEqBHT/g of extract)**
Fruits	5.62	8.17 ± 1.3	2.77 ± 0.4	0.12 ± 0.01	120 ± 2.4
Leaves	5.78	32.25 ± 0.9	2.09 ± 0.7	0.11 ± 0.02	135 ± 1.5
Root bark	3.41	8.8 ± 1.8	2.58 ± 0.3	0.12 ± 0.00	137 ± 3.5
Root wood	3.20	6.60 ± 0.5	2.15 ± 0.9	0.12 ± 0.05	140 ± 5.3
Vitamine C		12.61 ± 1.3	5.03 ± 0.3	0.23 ± 0.04	210 ± 1.9

Values are expressed as means ± Standard error (n = 3).

**Figure 2:**
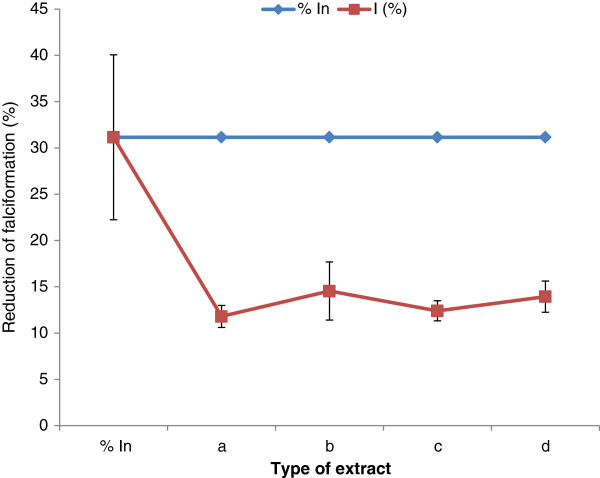
**Effect of extracts on the reversibility of the sickling; In: Initial percentage of falciformation; I: Percentage of falciformation after treatment with extract 250 μg/ml. (a)**: Fruits; **(b)**: Leaves; **(c)**: Root/bark; **(d)**: Root wood.

**Figure 3:**
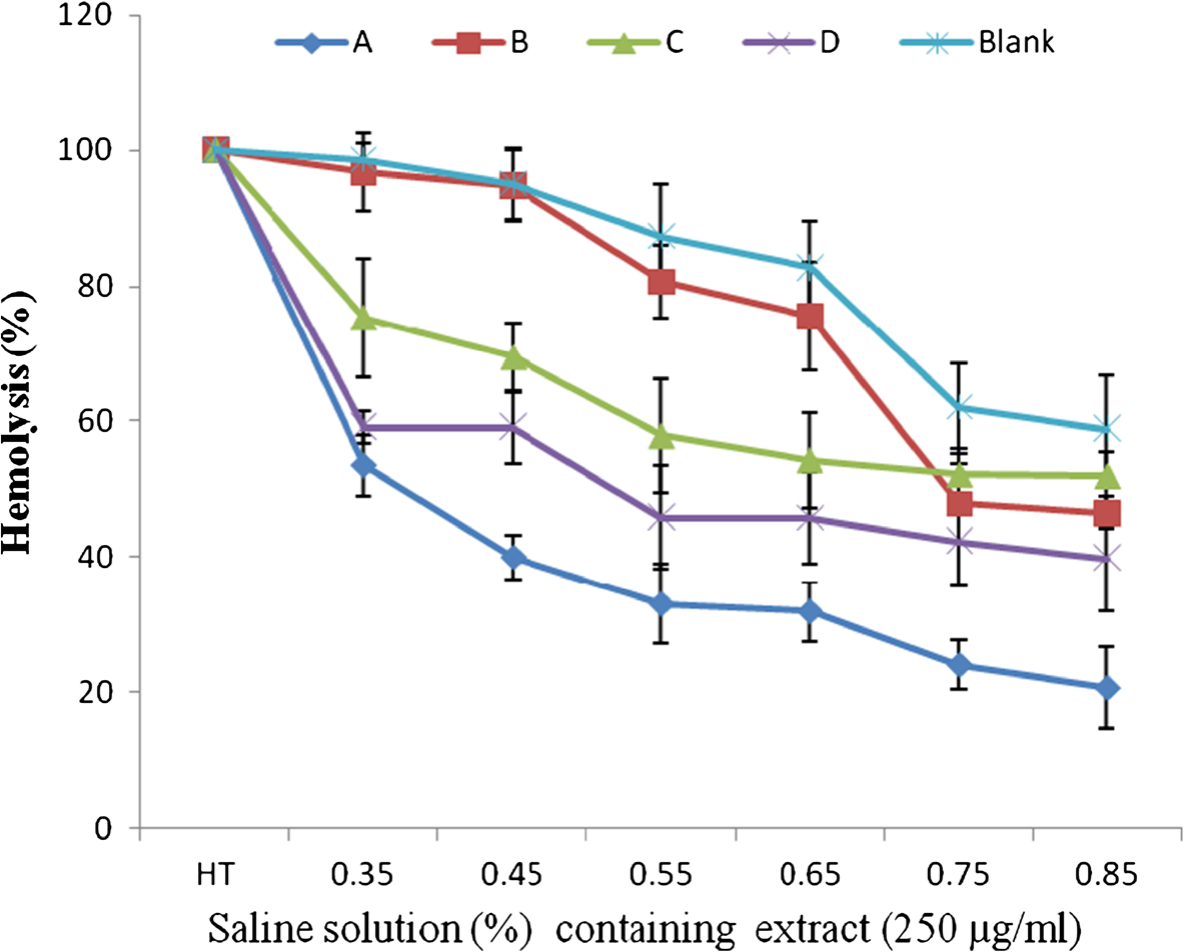
**Percentage of hemolysis of Sickling cells after treatment with 250 μg/ml of extract. (A)**: Fruits; **(B)**: Leaves; **(C)**: Root/bark; **(D)**: Root wood; HT: Total hemolysis.

After treatment of red blood cells with extracts at different concentrations (250, 500 and 1000 μg/mL), a significant decrease of the percentage of sickling cells was observed (Figure 1). This percentage of sickling varied from 38.78% at 1000 μg/mL to 44.05% at 250 μg/mL, depending on the part of the plant and the concentration of each extract used. The fruit extract demonstrated the highest decrease rate compared to others (Figure 1). The percentage of sickling cells increased with the extract concentration, although was non significant and non dose dependent for all extracts tested. The reversibility of the sickling cells was noted through its significant inhibition after 24 h of SS-RBC incubation with the extracts of *Z. heitzii* at 250 μg/mL. The average rate of this inhibition varied between 19.31 and 16.62% depending on the extract (Figure 2). The fruit extract demonstrated the highest decrease rate while the lowest was that of the leaf. Regarding the erythrocyte osmotic fragility, this result showed a significant decrease of the percentage of hemolysis while increasing the concentration of salt solution at 250 μg/mL of extracts (Figure 3). All the extracts tested showed a lower hemolysis percentage compared the control. Among the four extracts tested, that of the fruit presented the most significant reduction of heamolysis compared to extracts from other parts of the plant and then could have a better protective effect of the erythrocyte membrane.

The anti-radical and antioxidant activities of the extracts using different methods showed that these activities vary depending on the part of the plant. The IC_50_ of the extracts on DPPH and hydroxyl radicals varied from 6.60 to 32.25 μg/mL and from 9.2 to 3.5 μg/mL respectively (Table 1). All the extracts have significantly inhibited the hydroxyl radicals with IC_50_ less than 3 μg/ml except the control. The noticeable of the reducing power activity and the antioxidant properties of this plant could serve as a significant indicator of the antioxidant potential. The results of reducing power activity showed that at 50 μg/mL all the extracts demonstrated a weak activity (Table 1). The phytochemical screening of these extracts revealed the presence of alkaloids, phenols, mucilage and saponins. However, tannins and lipids were not detected in these extracts. Flavonoids are found in all extracts apart from that of the root.

## Discussion

For the pharmacological discovery of novel drugs, the primary essential information regarding the chemical constituents are generally provided by the qualitative phytochemical screening of plant extracts. In the present study, qualitative tests for different extracts of *Z. heitzii* showed the presence of several groups of bioactives molecules such as phenolics and alkaloids. Since the structure and chemical composition of plant parts affect the ability of permeability, infiltration and solubilization of groups of the bioactives compounds by the solvent, in the same way, the quantitative and qualitative differences between the phytochemical compounds in the extracts influence their biological activities. The treatment of erythrocytes with metabisulphite (2%) showed a significant augmentation of sickling. Thus sodium metabisulphite creates hypoxic conditions for red blood cells leading to the loss of the morphology and sickled erythrocytes. In vitro deoxygenation of RBC by sodium metabisulphite caused progressive aggregation and polymerization of the individual hemoglobin molecules [24]. The process of gelation (polymerization) of hemoglobin molecules increases the formation of sickling cells. The sickle cell hemoglobin (HbS) is a product of a defective genetic code of hemoglobin molecule. The sickling cells treated with extracts at different concentrations decreased as well as the naïve red blood cells used in the reversibility study when treated at 250 μg/mL. The activity of the extracts could be due to the presence of some bioactive compounds they possess. The phytochemical screening of these extracts revealed compounds such as phenols, flavonoids, alkaloids, saponins. The anti-sickling activity could be linked to their ability either to inhibit in vitro polymerization of haemoglobin or to some structural modification linked to the environment of haemoglobin by the extracts [25].

Several researches have established that the capability of a biomolecule to avoid in vitro polymerization depends on one or combinations of the following options: (a) The tendency and efficiency to bind to the complimentary contact region/site of deoxyHbS monomers [25,26]; (b) Modification of amino acid residues that contribute to the three-dimensional structures of HbS contact region and other critical sites [27]; (c) Stabilization of the R (relaxed) state of HbS molecule [3,24,27]. For sickle cell disorder, the study of antioxidants especially in various antisickling agents has a great importance because different antisickling agents have different degrees of effect.

Studies demonstrated that antioxidant molecules were found to be potent inhibitors of sickle cell haemoglobin polymerization, and equally improved the oxidant status of sickle erythrocytes [28-30]. This difference between the anti-sickling activities noted in our study could be attributed to different concentrations of the antioxidant molecules such as polyphenol, flavonoids identified in the different parts *Z. heitzii*. Also, the affinity of the molecule with haemoglobin binding site would determine the degree of the active anti-sickling agents. Like many proven anti-sickling agents, the most probable site for binding is the heme pocket and the erythrocyte membrane. The relation between antiradical and antioxidant activities of the extracts and their antisickling activity could be explained by the ability of these extracts to give hydrogen or electron atom to the iron molecule of haemoglobin [31]. Antioxidants (scavengers of free radicals) are believed to be major components of the anti-sickling properties [32]. Plants which demonstrated antioxidant property can be therapeutically useful [33]. Thus, it is believed that the higher the antioxidant property of an antisickling agent, the higher its possible anti-sickling effect, as this enables it reduce oxidative stress that contributes to sickle cell crisis and increase the membrane protection of the cells. Since the sickle cell anemic individual undergoes oxidative stress at the onset of crises, these plants can be used to mop up any free radical released and so contribute to the effective management of the disorder. The antioxidant properties of phenolics are important in scavenging and neutralization of free radicals which can cause damage to cells if left in the system. Thus the increased intake of dietary antioxidants from *Z. heitzii* fruits may help to maintain an adequate antioxidant defense status and consequently contribute to the management of SCD.

## Conclusion

The fruit extract of *Z. heitzii* demonstrated significant antisickling and antioxidant properties. Therefore further studies are indicated to establish these data in transgenic mice models for human SCD. In addition, the toxicity profiles and bioguided fractionation studies need to be undertaken.

## Competing interests

The authors declare that they have no competing interests.

## Authors’ contributions

NP carried out the study; MB and AMV assisted to the manipulation; BNPC assisted the manipulation, carried out the statistical analysis and prepared the manuscript; PCA initiate the research, prepared the manuscript and co-directed the research work with NYJ who supervised and provided reagents. All the authors read and approved the final manuscript.

## Pre-publication history

The pre-publication history for this paper can be accessed here:

http://www.biomedcentral.com/1472-6882/13/162/prepub

## References

[B1] WHO Managment of haemoglobin disorders Report of a joint WHO-TIF MEETING 2007 Nicosia, Cyprus 16 18 http://www.who.int/genomics/WHO-TIF_genetics_final.pdf

[B2] Mpiana PT Mudogo V Tshibangu DST Kitwa EK Kanangila AB Lumbu JBS Ngbolua KN Atibu EK Kakule MK Antisickling activity of anthocyanins from *Bombax pentadrum*, *Ficus capensis* and *Ziziphus mucronata*: Photodegradation effect J Ethnopharmacol 2008 120 3 413 418 10.1016/j.jep.2008.09.012 18930798

[B3] Ibraheem NK Ahmed JH Hassan MK The effect of fixed oil and water extracts of *Nigella sativa* on sickle cells: an in vitro study Singap Med J 2010 51 3 230 234 20428745

[B4] Aslan M Thornley-Brown D Freeman BA Reactive species in sickle cell disease Annal N Y Acad Sci 2000 899 375 391 10.1111/j.1749-6632.2000.tb06201.x 10863554

[B5] Imaga NOA The use of phytomedicines as effective therapeutic agents in sickle cell anemia Scientific Res Essays 2010 5 24 3803 3807

[B6] Okpuzor J Olumide A Ogbunugafor H Ifeanyi A The potential of medicinal plants in sickle cell disease control: a review Int J Biomed Health Sci 2008 4 2 47 55

[B7] Sofowora EA Isaac-Sodeye WA Ogunkoya LO Isolation and characterisation of an antisickling agent from *Fagara zanthoxyloides* root Llyodia 1975 38 2 169 71 1134214

[B8] Adesina SK The Nigerian *Xanthoxylum*; chemical and biological values AJTCAM 2005 2 3 282 301

[B9] Ouattara B Jansen O Angenot L Guissou IP Frederich M Fondu P Tits M Antisickling properties of divanilloylquinic acids isolated from *Fagara zanthoxyloides* Lam. (Rutaceae) Phytomed 2009 16 125 129 10.1016/j.phymed.2008.10.013 19110407

[B10] Cronquist A The evolution and classification of flowering plants 1988 New York: The New York Botanical Garden

[B11] Heywood VH Les plantes à fleurs 1996 Paris: Nathan

[B12] Tchiégang C Mbougueng PD Chemical composition of spices used in the preparation of Nah poh et du Nkui of West Cameroon Tropicultura 2005 23 4 193 200

[B13] Zirihi GN Mambu L Guede-Guina F Bodo B Grellier P In vitro antiplasmodial activity and cytotoxicity of 33 West African plants used for treatment of malaria J. Ethnopharm 2005 98 281 285 10.1016/j.jep.2005.01.004 15814260

[B14] Mbaze LM Poumale HM Wansi JD Lado JA Khan SN Iqbal MC Ngadjui BT Laatsch H Alpha-Glucosidase inhibitory pentacyclic triterpenes from the stem bark of Fagara tessmannii (Rutaceae) Phytochem 2007 68 5 591 595 10.1016/j.phytochem.2006.12.015 17270224

[B15] Meva’a ML Lado JL Wansi DJ Chieh ST Chiozem DD Mesaik AM Muhammad IC Oxidative burst inhibitory and cytotoxic amides and lignans from the stem bark of *Fagara heitzii* (Rutaceae) Phytochem 2009 70 1442 1447 10.1016/j.phytochem.2009.08.007 19747699

[B16] Jiofack TR Zanthoxylum heitzii (Aubrev. & Pellegr.) P.G. Waterman 2008 http://database.prota.org/dbtw-wpd/exel/dbtwpub.dll?AC=QBE

[B17] Mokondjimobe E Miantezila BJ Sabri B Dzeufiet PD Chenal H Otsudi’andjeka JB Bipolo S Besse M Mamadou G Nzouzi NL Kamtchouing P Meddah B Okpwae OJ Schobiltgen F Eto B Fagaricine, a new immunorestorative phytomedicine from *Zanthoxylum heitzii:* Preclinical and multicenter cohort clinical studies based on HIV-infected patients in six countries Phytopharmacol 2012 2 1 26 45

[B18] Jaja SI Kehinde MO Gbenebitse S Mojiminyi FBO Ogungbemi AI Effect of vitamin C on arterial blood pressure, irreversible sickled cells and osmotic fragility in sickle cell anemia subjects Nig J Physio Sci 2000 16 1–2 14 18

[B19] Joppa KM Vovor A Eklu-Gadegbeku K Agbonon A Akikokou K Gbeassor M Effet de Morinda lucida benth. (Rubiaceae) et de Newbouldia leavis p. beauv. (bignoniaceae) sur la falciformation Med Trop 2008 68 251 256 18689316

[B20] Oyaizu M Studies on products of browing reaction: anti-oxydative activities of products of browing reaction prepared from glucosamine Jap J Nutr 1986 44 307 315 10.5264/eiyogakuzashi.44.307

[B21] Mensor L Menezes F Leitao G Reis A Dos Santos T Coube C Leitão SG Screening of Brazilian plant extracts for antioxidant activity by the use of DPPH free radical method Phytother Res 2001 15 27 130 10.1002/ptr.687 11268111

[B22] Benzie F Strain J The ferric reducing ability of plasma (FRAP) as a measure of antioxidant power: the FRAP assay Anal Biochem 1996 239 70 76 10.1006/abio.1996.0292 8660627

[B23] Trease GE Evans WC Pharmacognosy. Bailliere Tindal, London: Urquiaga I, Leighton F: Plant polyphenol antioxidants and oxidative stress Biol Res 2000 33 9716 9760

[B24] Chikezie CP Sodium metabisulfite–induced polymerization of sickle cell hemoglobin incubated in the extracts of three medicinal plants (*Anacardium occidentale, Psidium guajava,* and *Terminalia catappa*) Pharmacognosy Mag 2011 7 26 126 132 10.4103/0973-1296.80670 PMC3113351 21716622

[B25] Bianchi N Zuccato C Lampronti I Borgatti M Gambari R Fetal Hemoglobin Inducers from the Natural World: A novel approach for identification of drugs for the treatment of β-Thalassemia and sickle-cell anemia eCAM 2007 6 2 141 151 18955291 10.1093/ecam/nem139 PMC2686630

[B26] Abdulmalik O Safo MK Chen Q Yang J Brugnara C Abraham DJ O-FK Asakura T 5-hydroxymethyl-2-furfural modifies intracellular sickle haemoglobin and inhibits sickling red blood cells Brit J Haematol 2005 128 552 561 10.1111/j.1365-2141.2004.05332.x 15686467

[B27] Oyewole OI Malomo SO Adebayo JO Comparative studies on antisickling properties of thiocyanate, tellurite and hydroxyurea Pak J Med Sc 2008 24 18 22

[B28] Stuart J Mojiminiyi FB Stone PC Culliford SJ Ellory JC Additive in vitro effects of antisickling drugs Brit J Haematol 1994 86 820 830 10.1111/j.1365-2141.1994.tb04836.x 7918079

[B29] Imaga NO Shaire EA Ogbeide S Akindele SK In vitro biochemical investigations of the effects of *Carica papaya* and *Fagara zanthoxyloides* on antioxidant status and sickle erythrocytes Afr J Biochem Res 2011 5 8 226 236

[B30] Nwaoguikpe RN Braide W The antisickling effects of some micronutrients and antioxidant vitamins in sickle cell disease management Med Medic Sc J 2012 3 5 334 340

[B31] Kasi P Natarajan S Periyanaina K Shanmugaiahthevar K Bioprotective properties of seaweeds: in vitro evaluation of antioxidant activity and antimicrobial activity against food borne bacteria in relation to polyphenolic content Compl Altern Med 2008 8 2 11 10.1186/1472-6882-8-2 PMC2475528 18613983

[B32] Tatum VL Chow CL Antioxidant status and susceptibility of sickle erythrocytes to oxidative and osmotic stress Free Radical Res 1996 25 2 133 139 10.3109/10715769609149918 8885331

[B33] Kanatt S Chander R Sharma A Antioxidant potential of mint (*Mentha spicata* L.) in radiation- processed lamb meat Food Chem 2007 100 451 458 10.1016/j.foodchem.2005.09.066

